# Neutrophil (dys)function due to altered immuno-metabolic axis in type 2 diabetes: implications in combating infections

**DOI:** 10.1007/s13577-023-00905-7

**Published:** 2023-04-28

**Authors:** Pooja Yedehalli Thimmappa, Sampara Vasishta, Kailash Ganesh, Aswathy S Nair, Manjunath B Joshi

**Affiliations:** Department of Ageing Research, Manipal School of Life Sciences, Manipal Academy of Higher Education, Planetarium Complex, Manipal, Karnataka 576104 India

**Keywords:** Metabolism, Infections, Neutrophils, Type 2 diabetes, Neutrophil extracellular traps, Immuno-metabolism

## Abstract

Metabolic and inflammatory pathways are highly interdependent, and both systems are dysregulated in Type 2 diabetes (T2D). T2D is associated with pre-activated inflammatory signaling networks, aberrant cytokine production and increased acute phase reactants which leads to a pro-inflammatory ‘feed forward loop’. Nutrient ‘excess’ conditions in T2D with hyperglycemia, elevated lipids and branched-chain amino acids significantly alter the functions of immune cells including neutrophils. Neutrophils are metabolically active cells and utilizes energy from glycolysis, stored glycogen and *β*-oxidation while depending on the pentose phosphate pathway for NADPH for performing effector functions such as chemotaxis, phagocytosis and forming extracellular traps. Metabolic changes in T2D result in constitutive activation and impeded acquisition of effector or regulatory activities of neutrophils and render T2D subjects for recurrent infections. Increased flux through the polyol and hexosamine pathways, elevated production of advanced glycation end products (AGEs), and activation of protein kinase C isoforms lead to (a) an enhancement in superoxide generation; (b) the stimulation of inflammatory pathways and subsequently to (c) abnormal host responses. Neutrophil dysfunction diminishes the effectiveness of wound healing, successful tissue regeneration and immune surveillance against offending pathogens. Hence, Metabolic reprogramming in neutrophils determines frequency, severity and duration of infections in T2D. The present review discusses the influence of the altered immuno-metabolic axis on neutrophil dysfunction along with challenges and therapeutic opportunities for clinical management of T2D-associated infections.

## Introduction

Nutrients and metabolites significantly regulate effector functions of innate immune cells in both steady state and during infections. Bidirectional crosstalk between the innate immune system and metabolic pathways form an immuno-metabolic axis that is intricately regulated. Accordingly, metabolic diseases such as obesity, type 2 diabetes (T2D) and non-alcoholic fatty liver disease are characterized by chronic low-grade inflammation with elevated pro-inflammatory mediators that alter innate immune functions and overt into a feed-forward loop leading to excessive inflammation [1]. Neutrophils are metabolically active and effector functions carried out by these cells are energy dependent [2]. Neutrophils constituting about 60–70% of white blood cells are the first non-local immune cells to respond to both inflammatory or infectious stimuli and thus making these cells as the first line of defense [3, 4]. During physiological conditions, neutrophils are generated through ‘steady state granulopoiesis’, where about 10^11^ neutrophils per day are released from bone marrow and this process is regulated by a master transcription factor CEBPα in association with chemokine axis, adhesion molecules and growth factors. Steady-state granulopoiesis shifts to ‘emergency mode’ during acute infections to increase neutrophil numbers which is driven by CEBPβ and associated with elevated levels of pro-inflammatory mediators including G-CSF [5]. Neutrophils are the short lived, fugitive, most abundant and terminally differentiated innate immune cells, eliminate infections through evolutionary conserved biological processes such as phagocytosis, degranulation, producing extracellular traps and regulating macrophages and B cell functions [3]. These processes rely upon glycolysis, stored glycogen, pentose phosphate pathway, TCA cycle intermediates and glutaminolysis for the source of energy [6].

Although neutrophils are attributed to their beneficial effects to eliminate infections, mounting evidences have shown that these cells display adverse effects associated with several diseases including T2D. Several labs including our own studies using pre-clinical and clinical models have demonstrated that hyperglycemia activates neutrophils constitutively and impedes their response to infections [7]. Hyperglycemia in T2D significantly reprograms neutrophil metabolism and reduces effector functions. As a consequence of elevated glucose concentrations in T2D, molecular shunting of glucose metabolism from glycolysis to polyol pathways is observed. In normoglycemic conditions, the glucose flux through the polyol pathway is limited due to low affinity and high Km (Michaelis constant) value of aldose reductase for glucose (50–100 mM) and hence, a major proportion of glucose is metabolized by hexokinase feeding into glycolysis [8, 9]. However, the excess glucose concentration triggers aldose reductase resulting in depleted Nicotinamide adenine dinucleotide phosphate (NADPH) levels, a reducing equivalent and subsequently, accumulates osmotically active sorbitol [10–12]. Interestingly, several studies have demonstrated that the km value of aldose reductase for glucose varies among tissues such as 70 mM for the human placenta [13], 0.15 mM in rat lens, 0.11 mM for bovine lens and 651 mM for muscle tissue [13]. Aldose reductase activity was three times higher in diabetic individuals in erythrocytes and a significant correlation was observed between the enzyme activity and sorbitol levels [14]. Our metabolomics analysis in neutrophils isolated from T2D individuals also showed increased sorbitol levels [15]. These changes lead to reduced availability of NADPH for the normal functioning of neutrophils there by decreasing levels of the intracellular ROS scavengers, glutathione and modifies transcription factors activating pro-inflammatory genes (IL-6, TGF-α, TGF-β) [16].

Decreased scavenge and increased formation of cytokines activates naive neutrophils, causing a feed-forward loop of excessive inflammation in diabetes [17, 18]. Clinically, T2D subjects show increased pre-disposition to infections including sepsis, fungal infections, foot ulcers, bacterial pneumonia, urinary tract infections, blood stream infections, skin infections, soft tissue and eye infections. Interestingly, metabolic health of an individual determines the frequency, duration and severity of the infections. Nutrient ‘excess’ condition in T2D characterised by hyperglycemia, elevated lipids and branched-chain amino acids significantly alter immuno-metabolic axis, there by leading to constitutive activation, compromised mobilization and impeded acquisition of effector or regulatory activities of neutrophils and render these subjects for recurrent infections. In the present review, we catalogue and discuss how the altered immuno-metabolic axis in T2D influence neutrophil functioning during various infections. Further, we discuss challenges and opportunities to restore neutrophil function in T2D subjects for the clinical management of infections.

## Neutrophils reprogram their metabolism to carry out effector functions

Neutrophils are metabolically active cells and rely on distinct metabolic pathways for their energy need. The neutrophils contain a modest number of mitochondria which makes them rely on other sources of the metabolic processes for their effector functions [2]. During differentiation, neutrophils utilize larger proportions of energy from glycolysis and FAO-mediated mitochondrial respiration and after being released into circulation, upon encountering harsh environment such as acute inflammation and infections, with the inaccessibility of glucose these cells adapt to glycogenolysis [19]. However, in hypoxic conditions, neutrophils shunt to glycolysis rather than mitochondrial respiration [20]. Neutrophils perform diverse immunological functions including ROS formation, phagocytosis, degranulation and extracellular trap formation to immobilize and eliminate pathogens. Even though glycolysis is the fundamental metabolic process, under glucose-depleted conditions, neutrophils depend on glycogenolysis for functions including phagocytosis [20]. Primed/activated neutrophils express increased levels of GLUT receptors on their surfaces associated with increased glucose uptake [13]. Rodríguez-Espinosa et al., demonstrated the metabolic requirement of NETs formation where, chromatin condensation was glucose independent and however, glucose was required for chromatin release during NETosis [21]. Primarily neutrophils depend on glycolysis as an energy source for NETs production. Neutrophils treated with a hexokinase inhibitor, 2-deoxyglucose (2-DG) reduced NETs formation in response to IL-6 and glucose [7].

Neutrophils are only myeloid cells that are competent in gluconeogenesis and glycogenesis, where these cells convert glucose-1-phosphate to glucose-6-phosphate, which is hydrolysed to glucose by glucose-6-phosphatases (G6Pase) which serves as a main source of ATP [22–24]. Robinson et al., showed an increased accumulation of glycogen in neutrophils that were isolated from inflammatory exudates in the peritoneal cavity of guinea pigs’ inflammation site compared to peripheral neutrophils [25]. In spite of limited oxygen and metabolic substrate, neutrophils survive and perform their functions in infected and injured tissue. A recent study showed that neutrophils undergo dynamic metabolic adaptation with a net increase in glycogen generation and storage by activating metabolic pathways gluconeogenesis and glycogenesis for their survival and effector functioning in infected sites. Further, authors demonstrated that neutrophils regulate glycogenesis and also utilize non-glucose substrates to generate glycogen stores by using radioactive flux and LC–MS tracing of U-^13^C glucose, glutamine, pyruvate and U-^14^C glucose in LPS treated or altitude-induced hypoxia in neutrophils [22].

An additional glucose-dependent metabolic pathway in neutrophils is the pentose phosphate pathway (PPP) also known as hexose monophosphate shunt which has been observed in both activated and resting neutrophils [26]. PPP is also involved in NETs formation induced by PMA and AF, which was demonstrated by blocking glucose-6-phosphate dehydrogenase of PPP by adding 6-aminonicotinamide (6-AN) [26]. For the synthesis of ROS, neutrophils switch to PPP to produce NADPH for superoxide generation which is catalysed by NADPH oxidase in phagosomes. Mutations in genes coding for subunits of the NADPH complex fail to produce ROS and leads to insufficient production of NETs which subsequently manifests into chronic granulomatous disease (CGD) [27]. As an alternative to PPP, mitochondrial glutaminolysis supports ROS formation by contributing to the formation of NADPH [6]. Chemotaxis is a pre-requisite for neutrophils to combat infections. The energy required for the migration of neutrophils towards the chemoattractant is provided by ATP generated from purinergic signaling from the mitochondrial TCA cycle and glycolysis [19]. Furthermore, neutrophils adopt/activate fatty acid metabolism during limited glucose availability. Mitochondrial FAO converts fatty acids to acyl-CoAs then it enters to TCA cycle as acetyl-CoA, and energy in form of ATP is generated through the electron transport chain (ETC).

Studies have demonstrated the significant role of glutaminolysis as a source of energy in neutrophils in performing their effector functions. Under glucose-depleted conditions cells including neutrophils, glutamate undergoes glutaminolysis and form α-ketoglutarate to enter the citric acid cycle and subsequently makes malate and further transform to pyruvate [28]. Using rat models, neutrophils displayed higher consumption and utilization of glutamine than glucose [29]. Glutamine has also been shown to play a significant role in the regulation of NADPH oxidase in rat neutrophils. Glutamine elevated the expression of gp91, p22 and p47 subunits of NADPH oxidase and generated increased super oxides [170]. Neutrophils from Wistar rat showed maximum uptake of glutamine when cultured in glutamine-rich media [28] and utilized energy for antimicrobial activity [30]. Furukawa et al., 1997 in post-operative subjects found decreased levels of glutamine and further showed that neutrophils from these subjects upon culturing with glutamine showed increased bactericidal activity [31]. Subsequently, the same group showed glutamine supplementation increased the ability of neutrophils from post-operative patients to perform efficient phagocytosis and produce elevated levels of reactive oxygen species [171]. Neutrophils display defective bacterial killing when gluconeogenesis and glutaminolysis are disrupted. Glutaminolysis plays a major role in glycogen synthesis in neutrophils. Glycogen levels were reduced in neutrophils stimulated with LPS in the presence of glutaminase/glutaminolysis inhibitor BPTES and MB05032 [22]. Taken together, these studies indicate glutamine plays an important role in regulating the effector functions of neutrophils.

## Influence of hyperglycemia-induced inflammation on over-functioning of neutrophils

Precise neutrophil recruitment to infected tissue/organ is very important to combat microbes and to restore immune homeostasis during inflammation modulation and resolution, wound healing and tissue repair. Indeed, subjects with reduced absolute neutrophil counts are more prone to repeated infections while uncontrolled/abnormal neutrophil function may lead to tissue damage and associated autoimmune disorders [33]. Over the years, studies have demonstrated that in T2D, hyperglycemic milieu affects the normal functioning of neutrophils. Tian et al., in 2016, showed that exposure to advanced glycation products diminished neutrophil viability, accelerated cellular apoptosis, and hindered neutrophil migration [34. Neutrophils upon exposure to AGEs showed an increase in the production of inflammatory mediators and oxidative stress. However, no morphological changes were observed in neutrophils in T2D subjects [35]. Hyperglycemia impaired neutrophil mobilization and led to an enhanced metastatic spread in cancer [36]. Kuwabara et al., 2018 treated bronchoalveolar (BAL) tissue of Goto-Kakizaki (GK) and High Fat Diet (HFD) mouse with LPS and demonstrated an impaired in the chemotactic property of neutrophils, decrease in the neutrophil count, reduced release of IL-1β, IL-6, TNF-α and MPO activity along with an increase in CXCL3 levels. These results revealed impaired response of neutrophils to LPS in HFD mouse  [37]. Proteins such as Phospho-IKBα, phospho-NFκB and NFκB involved in the activation of TLR4 pathway in neutrophils were decreased in LPS-treated BAL of HFD-fed mice suggesting neutrophils from diabetic mouse were LPS insensitive [37]. In T2D, degradation of the extracellular matrix by proteases was overruled even in the presence of protease inhibitors indicating accelerated activity of proteases in T2D. Protease isoforms of membrane bound and intracellular cathepsin B and leukocyte elastase were significantly increased in T2D conditions [38]. Platelet activation plays an important role in process of atherogenesis and thrombosis in T2D-associated myocardial ischemia. Neutrophils in hyperglycemic conditions are triggered to produce S100 calcium-binding protein A8/A9 which binds to the receptors of Kupffer cells to enhance the production of thrombopoietin, which in turn interacts with c-MPL receptor on megakaryocytes and bone marrow progenitor cells to increase the proliferation resulting in reticulated thrombocytosis [39]. Umsa-ard et al., 2015 showed that hyperglycemia increased the expression of CD11b and CD66b in neutrophils which in turn induced the adherence of neutrophils to endothelial cells, may or at least in part involved in the development and progression of atherosclerosis in diabetic subjects [40]. Comparative transcriptome analysis of T2D and normal neutrophils deciphered significant differential expression of nearly 50 genes related to inflammation and lipid metabolizing genes including *SLC9A4, NECTIN2, LILRB5*, *AKR1C1* and *PLPP3* [41]. Methylglyoxal, a metabolite observed significantly higher in T2D subjects stimulated neutrophils to release cytokines such as IL-6, TNF-α and IL-8 rendering neutrophils to a pro-inflamed condition which may lead to reduced response to infections [42]. Bcl-2 is an anti-apoptotic protein and Bax is a pro-apoptotic protein. In T2D, significant apoptotic changes are seen where Bax expression is comparatively higher than Bcl-2 indicating increased apoptotic neutrophils [43]. Microarray analysis deciphered differential expression of miRNAs in neutrophils isolated from diabetic skin wound in comparison with non-diabetic derived neutrophils, particularly *miR-129-2-3p*. This miRNA regulates Ccr2 and Casp6 translation and is involved in inflammatory responses, phagocytosis, apoptosis, endocytosis, chemotaxis and endocytosis in neutrophils. The deregulation of *miR-129-2-3p* contributed to the dysfunction of diabetic-derived neutrophils [44]*.*

### Diabetic microenvironment impedes phagocytic ability in neutrophils

Phagocytosis is a central function of neutrophils to eradicate pathogens during infections and this key process is altered in T2D. The process of phagocytosis involves proteins such as cathepsin, defensin, lactoferrin and lysozyme to kill the pathogens [45]. Neutrophil apoptosis regulates effector functions, longevity, and free radical-mediated injury. Neutrophil-mediated phagocytosis is an effective immune function in *Mycobacterium tuberculosis* infections [46]. The major metabolic product of gut microbiota are short-chain fatty acids such as butyrate, propionate and formate. Increased levels of short-chain fatty acids (SCFAs) cause decreased neutrophilic mycobacterial phagocytosis along with decreased production of superoxide, hydrogen peroxide and hypochlorous acid. Due to altered levels of SCFAs, T2D confers a threefold increased risk for the development of tuberculosis [46]. T2D is the highly associated factor responsible for the complication of septic endophthalmitis and correlated to *K. pneumoniae*-induced liver abscess. Neutrophil-mediated phagocytosis of capsular serotypes K1/K2 of *K. pneumoniae* was lower in patients with T2D than normal healthy controls. Poor glycemic control in endophthalmitis and meningitis was associated with a decreased phagocytic rate of *K. pneumoniae* [47]. This defective killing of K1 and K2 strains was compensated by NETs-mediated killing [48]. Davidson et al., (1984) showed that phagocytic impairment in neutrophil was partially due to the reaction between the plasma protein and glucose concerned with opsonisation [49]. *Staphylococcus aureus* induced phagocytic activity was decreased in diabetic subjects in comparison with control after both the groups were treated with nicotinamide [50]. Mazade et al., 2001 demonstrated that in T2D, neutrophil-mediated phagocytosis of type 3 group *B. Streptococcus* was impaired. Authors showed that upon using alrestatin which is an inhibitor of the aldose reductase pathway, superoxides were generated for a significant increase in phagocytosis of GBS [51]. Adiponectin has been shown to reduce the production of ROS and also inhibited the process of phagocytosis. Adiponectin reduced the binding of *E. coli* bacteria to the surface of bacteria by reducing the complement receptor Mac-1 and further inhibited the phosphorylation of PKB and ERK1/2 to reduce the phagocytic process [52]. Neutrophil functions require ATP-as an energy source, which is produced mainly by the metabolism of glucose to lactate. As neutrophils from diabetic hosts display impaired glucose metabolism, the reduced energy of neutrophils in diabetic hosts may render them functionally refractory [53]. Taken together, T2D subjects are extensively prone to infections due to the defective phagocytic function and elucidating pathways to re-activate phagocytosis may be important to maintain homeostasis of the innate immune system.

### T2D neutrophils form constitutive NETs and renders to reduced response to infections

Upon activation, neutrophils expel their DNA and granular proteins to form a web like structure known as Neutrophil Extracellular Traps (NETs). Highly activated neutrophils produce NETs through which the pathogens are trapped and eliminated [45]. NETs consist of DNA to which histones and proteins released from granules are bound [54]. NETs immobilize the pathogens, preventing pathogens from spreading and also facilitates phagocytosis of the captured pathogen [4]. T2D is associated with increased levels of pro-inflammatory cytokines such as TNF-α, IL-6 and IL-8 and which leads to the constitutive activation of NETs. Earlier studies from our lab have shown that hyperglycemic conditions in T2D induced constitutive NETosis and further neutrophils failed to form NETs in response to LPS [7]. Impaired or excessive NETosis play a role in promoting inflammation, thrombosis and endothelial dysfunction which contribute to diabetic complications [55]. It has been shown that elevated levels of homocysteine in T2D as a potent inducer of NETs. Mechanistically, NETs formed by homocysteine varied from other inducers by their requirement for calcium flux and mitochondrial superoxide [56]. PMA is a potent inducer of NETs, glucose ability to mimic PMA to induce NETs was related to its effect on PKC. Glucose also induced NADPH oxidase required by neutrophil for NETs formation [7, 55]. In T2D, neutrophils have a higher concentration of intracellular calcium and on the other hand, calcium flux is required for the formation of NETs. Increase in calcium flux elevated PAD4 levels which mediate histone citrullination [57]. It was observed that in T2D, neutrophils on treatment with IL-6, LPS and TNF-α did not form any extended NETs [7, 55, 58]. Miyoshi et al., demonstrated that serum MPO-DNA complexes associated with circulating NETs were significantly higher in T2D patients and suggested that elevated NETs formation in T2D patients may be a risk of microvascular complications. NETs formation is linked to both impaired wound healing and microvascular complications [59].

Degranulation is a process where neutrophils release their antimicrobial cytotoxic and other granular proteins from secretory vesicles. Azurophilic granules form first at different stages of neutrophil development, followed by specialised granules such as gelatinase granules, and finally secretory vesicles [60]. According to the formed-first-released-last hypothesis, these granules are easily mobilised upon an inflammatory stimulus at the plasma membrane in reverse order to their production [61]. Neutrophils produce a mixture of proteins from primary granules (azurophilic), secondary granules (specific) and tertiary granules, content of these granules has an antimicrobial function and help in eliminating infections. However, uncontrolled secretion of these mediators during the degranulation causes respiratory burst and leads to cell-mediated tissue damage [62]. Azurophilic granules constitute various peptides/protein including MPO, alpha-defensins, BPI, elastase, proteinase-3, and cathepsin G. Azurophilic granules constitute various peptides/protein includes alpha-defensins, MPO, elastase, cathepsin G, BPI, and proteinase-3. Small peptides such as alpha-defensins and cathelicidins play a role in the immune response by forming transmembrane pores that protect against a variety of fungi, bacteria, protists, and enveloped viruses. BPI neutralizes gram-negative bacteria by binding to the negatively charged LPS neutralizes the microbial activity [63]. Specific or secondary granules mainly constitute MMP, neutrophil collagenase-2, gelatinase-B, stromomelysin and leukolysin. Studies have shown that high glucose levels hinder neutrophil functions including degranulation. Hyperglycemia resulted in decreased *E. coli* endotoxin-induced neutrophil degranulation and an increase in coagulation [64]. Hyperglycemic conditions diminished inflammation-induced neutrophil degranulation and exacerbated procoagulant responses, whereas hyperinsulinemia inhibited fibrinolysis during the early inflammatory reaction due to extra stimulation of PAI-1 activity [65]. A Study showed reduced bacterial infections in diabetic mice with controlled blood glucose level [66]. Juan Huang et al., showed that high concentrations of plasma neutrophil elastase (NE) may also be considered as a marker of the development of complications, such as diabetic angiopathy and coronary artery disease [67]. Other studies showed that poor short‐term glycaemic and metabolic control in T1D patients were correlated with higher elastase concentration in plasma and neutrophils [68, 69].

### T2D is associated with an imbalance in redox homeostasis in neutrophils

Free radical formation and oxidative burst in neutrophils are one of the prime defense mechanisms to eliminate pathogens [70]. Higher levels of glucose and AGEs induce neutrophil activation and subsequently escalated oxidative stress via RAGE-ERK1/2 pathways. Ligation of RAGE and AGE potentially increases cytosolic ROS production via NADPH oxidase along with mitochondrial superoxide synthesis. Studies have demonstrated increased production of ROS (superoxide radicals, hydrogen peroxides) and Rreactive Nitrogen Species (NO, ONOO-) in neutrophils of T2D subjects in a resting state [70]. In T2D, neutrophils constitutively produce ROS at low levels and lose their ability to synthesize required levels of ROS in response to various stimuli. NADPH oxidase complex is a major source of ROS in neutrophils. PMA stimulation leads to the production of ROS through the activation of the protein kinase signaling cascade (PKC). P^47^phox is a cytosolic subunit and key protein in the assembly of NADPH oxidase. Triggering of a neutrophil by either PMA or fMLP leads to the phosphorylation of P47phox, a cytosolic subunit of NADPH complex and translocate to the plasma membrane to interact with flavocytochrome b558 [71–73]. Omori et al., 2008 stated that elevated glucose triggered ERK1/2-mediated premature translocation of the p47phox subunit of NADPH oxidase to the cell membrane, which resulted in constitutive superoxide production in neutrophils [74]. Oxidative burst (rapid release of reactive oxygen species) is controlled by inhibiting the action of ROS generating enzyme NADPH oxidase [70]. Hence, several attempts have been made to synthesize and explore NADPH oxidase inhibitors in reducing NETs. Decrease in the production of antioxidants such as catalase, SOD and Glutathione peroxidase in T2D also leads to the increased production of ROS [15]. Neutrophils are involved in the primary pathogenesis and progression of occlusive vascular disease due to lipid peroxidation and platelet aggregation through the production of ROS [75].

A vital component of the immune system, antimicrobial peptides (AMPs) are beneficial against a variety of pathogenic microorganisms, including fungi, bacteria, protists, and viruses [76]. AMPs communicate with inflammasomes and their complement systems, as well as pattern recognition receptors (PRRs) or chemokine receptors (CCRs), to establish a link between innate and adaptive immunity. AMPs are also involved in fundamental cellular functions including differentiation, proliferation, and apoptosis [172, 173]. The azurophilic and specific granules of the neutrophils are a rich source of AMPs which migrates to phagolysosomes and act on intracellular pathogens which are engulfed. AMPs are also released into extracellular space to kill microorganisms but also affect the other cells in the tissue. Human α-defensins are produced mainly by neutrophils; hence, these peptides are referred as human neutrophil peptides 1–4 (HNP-1, HNP-2, HNP-3, and HNP-4). HNP-4 is the least abundant and stored in PMN [77]. These peptides prolong their lives by preventing apoptosis, which enhances phagocytic activities. Host defence peptides (HDPs), on the other hand, may act as a “molecular brake” on macrophage-driven inflammation to optimize pathogen elimination with the least amount of negative consequences on surrounding tissues [78]. Cathelicidin levels in PTB-DM (pulmonary tuberculosis with diabetes) individuals are higher than TB, LTB (Latent tuberculosis), NTB and T2D alone individuals, this level is positively correlated with HbA1C level, bacterial burden and random blood glucose levels. AMPs appear to act as reliable and reproducible biomarkers for the therapeutic monitoring of TB-DM disease [79]. The diabetic patients exhibited increased plasma levels of HNP 1–3 (-defensin) than the healthy controls. This suggests that T2D promotes neutrophils to become constitutively activated. HNP1-3 may have clinical significance in diabetic patients with vascular or hypercholesterolemic dysfunction as it influences the LDL accumulation in the vasculature and inhibits fibrinolytic activity on the surface of vascular cells [80, 81]. The release of neutrophil extracellular traps also involves HNP 1–3. T2D is linked to low-grade inflammation, which produces aberrant inflammatory cytokines and NETs, which may be the primary cause of the elevated level of HNP-1 concentration in T2D participants. Nemeth et al., showed elevated levels of α-defensin (HNP1-3) in type 1 and type 2 diabetes, which were more pronounced during diabetic complications [82].

## Asynchronized metabolism in T2D leads to reduced response to infections

Diabetic individuals are highly susceptible to bacterial, fungal and viral infections. A wide spectrum of gram-negative/positive bacteria are associated with infections in T2D subjects (Table 1). Cellulitis is caused by *Staphylococcus aureus* and *Streptococcus pyogenes* [83]. *Streptococcus pyogenes or Clostridium spp.* are responsible for necrotizing fasciitis [83], whereas *Streptococcus pneumoniae*, *Mycoplasma pneumoniae*, *Chlamydia pneumoniae*, *Legionella spp.*, *Haemophilus influenzae*, *Staphylococcus aureus*, *Klebsiella pneumonia* and *Mycobacterium tuberculosis* cause community-acquired pneumonia [84]. Asymptomatic bacteriuria is a result of *Enterobacteriaceae* infection [85]. *Enterobacteriaceae*, *Staphylococcus saprophyticus*, *Enterococcus spp.*, rarely *Candida spp.* give rise to cystitis [86]. *Pseudomonas aeruginosa* infection reflects in necrotizing otitis externa. Rhinocerebral mucormycosis is caused by *Rhizopus* (> 90%), *Mucor* and *Absidia* species [87]. *Candida albicans* is associated with mucocutaneous candidiasis [88].

**Table 1:** Alterations in neutrophil functions in various infections

Infections	Pathogens	Perturbated neutrophil function	References
*Respiratory infections*			
• Pneumonia	*Streptococcus pneumoniae*	↓ NETs production, Impaired migration, ↓ Chemotaxis,	[155, 156]
• Influenza • H1N1	Influenza A Influenza B	↓ Neutrophil degranulation, impaired phagocytosis ↑ Infiltration of neutrophils	[156, 158]
• Tuberculosis	*Mycobacterium tuberculosis*	↓ Chemotaxis ↓ Phagocytosis	[156]
*Urinary tract infections*			
• Asymptomatic bacteriuria	Enterobacteriaceae* Escherichia coli* *proteus sp.*	↓ Chemotaxis	[156]
• Emphysematous phylonepritis	*E. coli, Entero bacter aerogenes, Klebsiella sp.,* *Proteus sp.,* *Candida,* *Streptococcus sp.,*	↓ Neutrophil recruitment	[157, 159]
• Pyelonephritis	*E. coli*, *Proteus sp.,*	Attenuated cytokine expression, Impaired neutrophil infiltration	[157]
• Emphysematous cystitis	*Enterobacteriaceae Staphylococcus saprophyticus*, *Candida sp.,*	↓ Neutrophil activity	[157]
• Perinephric abscess	*Gram negative bacilli*	Neutropenia	[157]
*Gastrointestinal and liver infection*			
• Gastritis	*Helicobacter pylori*	↑Infiltration of neutrophil ↑ Oxidative burst	[160, 161]
• Oral and esophageal candidiasis	*Candida albicans*	↓Super-oxide production	[162]
• Hepatitis C	Hepatitis C virus	↓ Neutrophil count	[163]
• Hepatitis B	Hepatitis B virus	↑ neutrophil–lymphocyte ratio	[164]
*Skin and soft tissue infection*			
• Foot infection	*S. aureus, S. pyogenes*, *Pseudomonas aerigunosa, anaerobes*	↓ Neutrophil phagocytic activity, ↓ Bactericidal activity	[157]
• Necrotizing fasciitis	*S. pyogenes* or *Clostridium sp*.	↓ Phagocytosis ↓Chemotactic activity	[157]
• Fournier gangrene	*E. coli, Klebsiella sp* *Proteus sp. Peptostreptococcus*	↓ Neutrophil function	[156]
*Head and neck infection*			
• Rhinocerebral mucormycosis	*Rhizopus, Mucor, Cunninghamella* *absidia species*	Impaired phagocytic response	[165]
• Periodontitis	*Actinobacillus (Haemophilus) actinomycetemcomitans*	↓ Chemotaxis ↓Phagocytosis	[156, 166]
• HIV	Human immune deficiency virus	↑Neutrophil apoptosis, Impaired Neutrophil phagocytosis and Chemotaxis	[167]

The primary fuel for neutrophils is produced as a result of glucose conversion to lactate [89]. Neutrophils also rely on glutamine and the oxidation of glucose [90]. T2D neutrophils display increased activity wherein these cells adhere to the endothelium and also migrate to the site of inflammation [91]. Neutrophils combat infections via chemotaxis [92], phagocytosis [93], and bactericidal responses [94]. These cells are associated with the production of reactive oxygen species [95]. In the diabetic *milieu*, excessive production of polyols and ketone bodies influencse the function of the neutrophils. In response to the *Candida* infection, neutrophil-induced phagocytosis was examined. Neutrophils when treated with the combination of high glucose (50 mM) and β-hydroxybutyrate (20 mM) had minimal ability to defend against the infection when compared with the controls. The study showed that the oxidative killing of *Candida* by neutrophils was inhibited due to high glucose and ketones. NADPH levels which are required for NETs formation are depleted as a result of a conversion of glucose to sorbitol in diabetic subjects [15]. Similarly, β-hydroxybutyrate is known to reduce the entry of glucose into the glycolytic pathway and favors the formation of the sorbitol and cause depletion in the NADPH levels which are vital for the neutrophils to fight against infections [96]. *Staphylococcus aureus*-induced respiratory infection in a hyperglycemic environment was inhibited by metformin in a db/db mice model. The study demonstrated that the number of neutrophils were significantly high in the bronchoalveolar lavage of the infected mice. Treatment with metformin activated the AMPK, which depletes the fuel needed for the growth of *Staphylococcus aureus.* This resulted in alleviating the infection [97]. T2D subjects are at high risk for *Burkholderia pseudomallei* infection which causes melioidosis. *Mycobacterium tuberculosis*-mediated tuberculosis infection is common in T2D subjects. PBMCs isolated from the diabetic subjects infected with *Burkholderia pseudomallei* and *mycobacterium tuberculosis* showed impaired IL-12p70 activity resulting in decreased production of IL-12. The reduced levels of IL-12 were correlated with low levels of glutathione (GSH) in diabetics. Treatment of the PBMCs with glutathione or N-acetylcysteine enhanced the combating activity of the neutrophils. Similarly, mice depleted with GSH were vulnerable to melioidosis. Hence, the study suggested that replenishing GSH will increase the innate immune function of diabetes [98]. The underlying signaling mechanism of GSH involves the decreased activity of γ-glutamylcysteine ligase by glucose. Conversion of glucose to sorbitol requires NADPH and the levels of the same are depleted in T2D. This NADPH serves as a cofactor for the regeneration of GSH. AGEs also deplete GSH by excess production of hydrogen peroxide and superoxide [12, 99–102]. Glycated bovine serum albumin has a lesser ability to bind to siderophores which provides more iron pool for the bacteria. This enables the bacteria to survive on the micronutrient and propagate the infection [103]. Uncontrolled activity of the neutrophils favors periodontitis in T2D. Individuals with obesity-induced T2D are prone to *Staphylococcus aureus*-mediated bone infection (osteomyelitis) after orthopedic surgery. Studies on the tibial wounds infected with *Staphylococcus aureus* in the mice model showed that infection levels were reduced after treatment with oligofructose and subsequently there was an increase in levels of gut *Bifidobacterium pseudolongum* which has an anti-inflammatory effect. Metabolic analysis of the ceacal and plasma of the T2D mice demonstrated an increase in spermine and spermidine levels and their supplementation impeded the bone infection in the mice model [104].

Esmann et al*.,* reported decrease in the glycolytic rate in the polymorphonuclear leukocytes isolated from subjects with uncontrolled diabetes [105]. This is due to the reduced activity of phosphofructokinase (PFK). The glycogen reserves and the rate of production of glycogen are decreased due to a reduction in the glycogen synthase activity. A phosphorylation cascade is activated because of the covalent modification of glycogen synthase. The glycogen synthase and glycogen phosphatase activity are hypothesized to be derailed in the leukocytes of diabetic subjects and this was restored after insulin treatment [105]. Neutrophils isolated from the streptozotocin-induced diabetic rats model demonstrated an impaired metabolic profile. The phagocytic activity and H_2_O_2_ production which was stimulated by PMA were reduced. The functional activity of glutaminase and G6PDH was reduced and that of PFK was increased in the diabetic neutrophils. The neutrophil function was restored after treatment with insulin [106]. Untargeted metabolomics in neutrophils isolated from T2D subjects demonstrated amelioration of 1-anhydrosorbitol and depletion of cysteinyl glycine. NADPH is used as a cofactor for three distinct pathways (a) 1-anhydrosorbitol production by aldose reductase, (b) synthesis of glutathione and (c) NETs formation and hence in T2D microenvironment leads to competition between these pathways for utilization of NADPH. Hence, in T2D conditions favor sorbitol formation and leaving insufficient pools of NADPH for the formation of glutathione and NETs formation in response to infections [15]. PMA and A23187 stimulated the formation of NETs via lactate production by increasing the activity of lactate dehydrogenase (LDH). Human neutrophils stimulated with exogenous lactate showed increased formation of the NETs. Treatment of neutrophils with sodium oxamate, an LDH inhibitor resulted in decreased formation of lactate and NETs in LPS induced sepsis model [107]. Our earlier studies have shown that homocysteine, a sulfur-containing amino acid induces NETosis in T2D subjects. We demonstrated that homocysteine constitutively elevated the levels of intracellular calcium and mitochondrial superoxides along with NETs formation in T2D conditions [56]. As a part of host retaliation against infections, neutrophils produce large amounts of intracellular superoxides. This will activate the nuclear factor NF-κB and develop a pro-inflammatory environment. Whereas proteasomal degradation of the NF-κB inhibitory subunit IκB-α, nuclear translocation of NF-κB and downstream activation of the pro-inflammatory environment was inhibited when bone marrow or peritoneal neutrophils are exposed to hydrogen peroxide [108]. Nicotinamide infusion improves neutrophil phagocytotic activity and oxidative burst in subjects with T2D [50]. PARP requires NAD which serves as its substrate. Nicotinamide reduces the PARP activity and also increases the NAD + NADH levels in the pancreatic beta cells, enhancing the activity of superoxide mutase which counteracts ROS [109]. Increased NETosis and PAD4 result in thrombotic [110] and inflammatory [111] complications associated with diabetes [112] and also delay the process of wound healing [57]. Infusion of 1 g/kg/day of l-arginine in diabetic rats alleviated wound healing. The levels of nitrite/nitrate and wound hydroxy proline which determines collagen synthesis were elevated as a result of l-arginine injection. Wound-breaking strengths were also enhanced after the supplementation of l-arginine [113]. Hyperglycemia favors the augmentation of the PFK and reduces the activity of glucose-6 phosphate dehydrogenase, and glutaminase. This hinders the pentose-phosphate pathway and subsequently neutrophil functions. G6PD deficiency causes a decrease in the production of O_2_(–) from the neutrophils thereby derailing its function [114]. Metformin, an anti-diabetic regulates various pathways to combat bacterial infections in T2D conditions. Mitochondrial respiratory-chain complex-1 was inhibited by metformin. It is also linked with the activation of the liver kinase B1 (LKB1)/AMPK pathway that enables innate immune response via neutrophil-mediated bacterial killing. Metformin also impeded neutrophil activation and improves the neutrophil–lymphocyte ratio [115] decreasing the levels of high sensitivity C-reactive protein, interferon-α(IFN-α) [116]. Metformin suppressed the folate cycle by inhibiting the dihydrofolate reductase [117], and utilization of the glycerol in Krebs cycle and gluconeogenesis by restraining the activity of bacterial glycerophosphate dehydrogenase resulting in decreased levels of dihydroxyacetone phosphate (DHAP) and elevated levels of nicotinamide adenine dinucleotide hydrogenase—nicotinamide adenine dinucleotide (NADH-NAD) ratio in the bacterial cells [118]. Metformin is known to activate adenosine 5'-monophosphate-activated protein kinase (AMPK) and subsequent improvement of neutrophil function enabled anti-inflammatory and bactericidal effects [119].

## Conclusion and future perspective

Recurrent infections in subjects with T2D are one of the major causes of increased mortality and morbidity. Impaired metabolic and exaggerated immunological responses cause chronic inflammatory milieu in T2D which leads to inefficient functioning of innate immune cells including neutrophils. Neutrophils (a) fails to respond to form extracellular traps; (b) show reduced phagocytic activity and (c) produce constitutive ROS in T2D due to significant cross-talk between metabolism and inflammation. Cellular and molecular mechanisms regulating the homeostasis of neutrophils during steady state (healthy condition) and emergency (infection) granulopoiesis in T2D is not known. Neutrophil homeostasis is regulated by a steady state and emergency granulopoiesis which are modulated by external stimuli such as inflammation and infections. The shift between these two states are dependent on the type, strength and duration of activation and thus impacts and reflects an individual’s metabolic health.

**Fig. 1:**
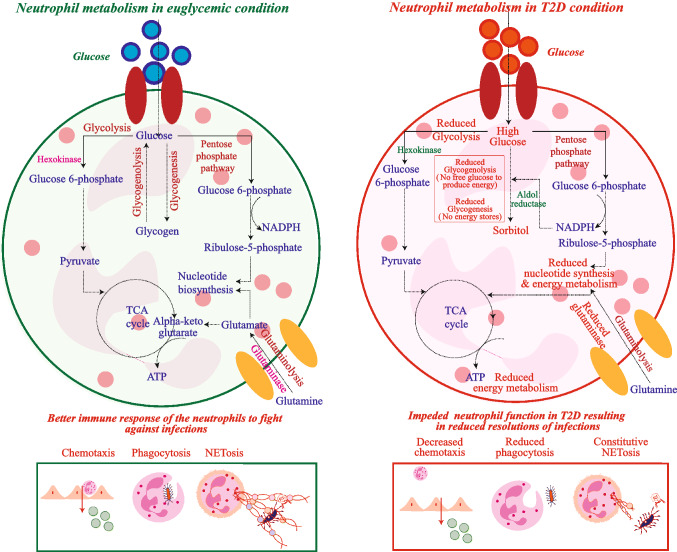
T2D microenvironment reprograms neutrophil metabolism and leads to reduced response to infections. Under euglycemic conditions, glucose is transported into neutrophils via GLUT1 and leads to the activation of various metabolic pathways. **A** Glucose is converted into pyruvate via glycolysis which, therefore, oxidizes into acetyl CoA and enters the TCA cycle which generates NADH & ATP. Further, the electron transport chain converts this NADH to ATP which serves as an energy source for the neutrophil activity. **B** Pentose-Phosphate Pathway (PPP) utilizes glucose-6-phosphate, an intermediate of the glycolytic pathway leading to the production of NADPH and Ribose-5-phosphate that are associated with redox activity and nucleotide biosynthesis. **C** Glutaminolysis: Glutamine via the activity of the glutaminase forms glutamate which is involved in the formation of α-ketoglutarate that enters the TCA cycle and is also associated with DNA and RNA synthesis. **D** Glycogenesis and glycogenolysis will maintain the optimum levels of glucose for the effective functioning of the neutrophils. All these 4 pathways produce sufficient energy and maintain redox activity in the neutrophils helping them in combating infections. In hyperglycemic conditions, the glucose is converted to sorbitol by consuming the NADPH and leads to oxidative stress. The glycogenolysis is reduced and therefore no free glucose is available for the production of energy sources. Reduced glycogenesis leads to deficits in the storage of glucose which serves as a source of ATP in the neutrophils. The glycolytic activity is also reduced in the neutrophils in the T2D condition. The activity of the glutaminase enzyme is also decreased leading to reduced nucleotide biosynthesis and energy metabolism. Overall, derailment of the major energy-producing pathways and altered redox activity disrupts the functional activity of the neutrophils making the T2D subjects susceptible to infections

Our present review discusses the breadth of prior research on immunometabolism in T2D to comprehend how neutrophil function is altered due to the reprogramming of metabolic pathways in diabetic conditions and to highlight therapeutic approaches to ameliorate aberrant neutrophil activity (Fig. 1). One potential strategy for the clinical management of infections associated with T2D is to restore neutrophil functions to respond to infections. Table 2 summarizes metabolic inhibitors and associated pathways in the context of neutrophil (dys)function in T2D. Studies, including our own lab, have shown that neutrophils were constitutively active in T2D subjects and showed reduced response to LPS/infections to form NETs [7]. Glycolytic reprogramming of innate immune cells involves multiple mechanisms. One of them is more rapid and relies on the translocation of pre-existing hexokinase II (HK-II) onto the outer mitochondrial membrane [120]. Hexokinases (HK) such as HK1, HK2 and HK3 are primary enzymes in glycolysis which is the main energy source for neutrophils to perform their functions. Matured neutrophils are enriched with hexokinase I and hexokinase II [121], however, during neutrophil differentiation of myeloid progenitors from cord blood (CB) and HSPCs gene for HK3 compared to HK1 and HK2 transcription. In a diseased condition like glycogen storage disease type Ib, neutrophils show defective glucose uptake and reduced levels of NADPH, G6P, ATP, lactate even though the expression of HK3, GLUT-1, HIF1-α expression were augmented [122]. Recent study showed that accumulation of 1,5-anhydroglucitol-6-phosphate (1,5-AG6P) which acts as an analog for G6PT and G6Pase-β inhibits hexokinase activity in GSD-Ib patients, thereby blocking the first step of glycolysis. Yeast strains that are deleted for hexokinase-2 (HXK2) was 2-DG resistant. Since Yeast growing on nonfermentable carbon sources are mainly dependent on glucose-phosphorylating enzymes GLK1 and HXK1 whereas yeast growing on glucose is predominantly dependent on HXK2 [123]. High glucose-induced NETs were inhibited by the inclusion of 2-DG, a synthetic analogue of glucose that precludes glycolysis by blocking hexokinase at very low concentration in culture media. Interestingly, 2-DG restored NETs formation in response to LPS under high glucose conditions [7]. Through cytokine and regulatory T cell (T-reg) mediated pathways, 2-DG exhibits anti-inflammatory properties. By increasing the production of cytokine production (IL-2) and preventing CTLA-4, a T-reg suppressor, 2DG therapy enhanced T-reg function [124, 125]. According to a study, 2-DG inhibited the PI3K/Akt pathway to reduce TNF production during the early stages of inflammation [126]. TLRs play a crucial role in controlling inflammatory signals. This TLR-induced acute and chronic inflammation was reduced by 2-DG as it blocks the glycolysis and ERK pathways and stops the inflammation process [127].

**Table 2:** Inhibitors of metabolic pathways to target neutrophil functions

Metabolic inhibitors	Pathways	Neutrophil functions	Reference
6-aminonicotinamide (6-AN)	6-phosphate dehydrogenase in pentose phosphate pathway (PPP)	Increases PMA-induced NETs formation	[26]
Alrestatin	Aldose reductase in Polyol pathways	Increases superoxide production, increases neutrophil phagocytosis	[51]
Sodium oxamate	Lactate dehydrogenase (LDH)	Decreases NETs formation induced by LPS	[108]
Metformin	Liver kinase B1 (LKB1)/AMPK pathway	Increases neutrophil-mediated bacterial killing	[116]
2-DG	Hexokinase in Glycolysis	Restore NETs formation in diabetes	[7]
Ranirestat	Aldose reductase in Polyol pathways	Reduce cytosolic ROS production and neutrophil elastase formation induced by high glucose	[15]
Cysteinyl glycine	Glutathione synthesis pathway	Decreases neutrophil elastase formation, cytosolic ROS production, and suppressed NETs formation in high glucose environment	[15]
Wortmannin and Diphenylene	NADPH oxidase	Attenuate PMA-induce NETosis	[140]
Itaconic acid (4-OI)	Nrf2/HO-1/Hif-1-dependent pathways	Reduces formation of NETs	[142]
Propofol	Inhibition of p-ERK and HOCl	Reduces PMA-induced NETs formation	[168]
Sivelestat	Neutrophil elastase	Prevent NETs formation induce by high glucose	[146]
Prostaglandin E2	Activates of the cAMP–PKA pathway	Inhibit NETosis by high glucose	[156]

Hence, inhibiting aldose reductase may facilitate in maintaining NADPH pools to utilize for forming NETs during infections. Earlier studies have elegantly demonstrated the kinetics aldose reductase reactions in lower and higher levels of its substrate glucose [128]. Under normal physiological conditions, about 3% of cytosolic glucose is processed *via* the polyol pathway, however, at higher concentrations of glucose, about 30% of the glucose enters the polyol pathway which makes it important in disposing of the glucose molecules and subsequent conversion to sorbitol [128]. Aldose reductase effectively catalyzes about 100 mM of D-glucose with a low Michaelis-Menton Constant, Km. This value is 20 times greater than the normal glycaemic level of 5 mM [129]. Accumulation of sorbitol results in elevated levels of reactive oxygen species, increased cellular damage and osmotic stress leading to diabetic complications [130]. Higher levels of ROS due to sorbitol may be one of the reasons for the constitutive production of NETs in hyperglycemic conditions [7].

Genetic variations in *ALR2* gene have been demonstrated in predisposition to the onset and progression of diabetic complications. Independent studies have shown that *ALR2* is activated by TNF-α [128, 129], synchrotron X-ray irradiation and oxidative stress during T2D leading to vascular damage [128]. Analysis of the transcription start site 2.1 kb upstream of the *ALR2* gene was studied in the Chinese population residing in Hong Kong who are diagnosed with non-insulin-dependent diabetes. The study revealed 7 alleles of *ALR2* of which (Z-2) was significantly associated with the early onset of retinopathy [131]. Abu-Hassan et al., performed a case–control study in the Jordanian population and revealed that C106T polymorphisms in the *ALR2* gene were associated with diabetic retinopathy [130]. A case–control study among the natives of the Bali region in Indonesia showed that C(-104)T polymorphism in the *ALR2* gene as a risk factor for diabetic retinopathy [132].

Targeting aldose reductase which drives the polyol pathway during diabetes could be a potential therapeutic strategy in the treatment and prevention of diabetic complications. A study by Varma et al. used quercitrin an isoflavone as aldose reductase inhibitors to prevent the accumulation of sorbitol formation in the cataract of diabetic patients [133]. Providing a Sorbinil-galactose diet proved to effectively abolish the polyol pathway of sugar metabolism, as evidenced by a progressive decrease in the lenticular dulcitol level and re-establishment of normal lens physiology in Sprague–Dawley rats [134]. Epalrestat (ONO-2235) and fidarestat (SNK-860) treatment were protective against diabetic nephropathy in clinical settings [135]. NADPH oxidase is required for glucose-formed NETs and its deficiency caused by aldose reductase’s competitive NADPH utilisation under high glucose conditions may be the cause of the impaired NET production. Ranirestat, a putative inhibitor of aldose reductase, also reduced cytosolic ROS and neutrophil elastase induced by high glucose. The formation of NETs was suppressed when neutrophils pre-treated with ranirestat under high glucose conditions. NADPH supplementation in neutrophil cultures in high glucose environments also markedly enhanced NET formation in response to LPS [15]. Additionally, two phase III clinical trials of the aldose reductase inhibitor ranirestat were completed successfully, and authors demonstrated its beneficial effects on diabetic neuropathy. The ranirestat therapy reduced the production of NETs by targeting aldose reductase activity and may serve as an effective method for preventing and treating cardiovascular problems in T2D [59]. Ranirestat treatment to streptozotocin (STZ)-diabetic rats and spontaneously diabetic Torii (SDT) rats showed inhibition of aldose reductase in both the sciatic nerve and lens [136, 137]. Another observational study by Ishibashi et al., demonstrated that comparatively to epalrestat, 500 nM ranirestat inhibited the effects of high glucose on elevated sorbitol levels, vascular cell adhesion molecule-1 mRNA levels in umbilical vein endothelial cells, and THP-1 cell adherence to human umbilical vein endothelial cells [138].

High glucose induces the formation of ROS and renders to increased oxidative stress. Using synthetic and natural anti-inflammatories may be another alternative to supress over functioning of neutrophils and NETs formation. A substantial drop in cysteinyl glycine, a crucial metabolic intermediate in the glutathione synthesis pathway, was observed in a metabolomic analysis of T2D neutrophils [15]. Glutathione supplementation effectively diminished glucose-induced neutrophil elastase and cytosolic ROS production and suppressed NETs formation in high glucose environment [15]. Inhibiting glucose-induced signalling changes and simultaneous activation of neutrophils to combat infections may be one of the potential approaches. The development of functional NETs may be aided by the combined autophagy and Nox2-dependent chromatin decondensation in intact neutrophils as well as the suppression of caspases. It has been shown that the PI3K/autophagy and NADPH oxidase inhibitors wortmannin and diphenylene iodinium (DPI), respectively, attenuated PMA-induced NETosis [139]. High glucose influences the phosphorylation of various upstream kinases, including AKT, ERK, and JNK (C-jun N terminal kinase). However, when neutrophils were precultured in high glucose and stimulated with LPS, these effects were abrogated. Newer insights into upstream kinases induced by glucose may aid in the development of therapeutic targets to block the effects of glucose and simultaneously restoring NETs in the presence of infections [140]. A metabolic regulator, itaconic acid (4-OI) blocked the Nrf2/HO-1/Hif-1-dependent pathways that lead to NET release. According to a study by Gabriela Burczyk et al., pre-treatment with 4-OI, a metabolic regulator, reduced the formation of NETs by increasing the expression/activation of Nrf2 and HO-1 and diminishing the expression of HIF-1, which was otherwise reduced and elevated by LPS, respectively, in mice's bone marrow-derived neutrophils [141]. It has been demonstrated that hyperglycemia reduces LPS-induced neutrophil degranulation, which in turn reduces the release of myeloperoxidase and elastase from azurophilic granules. This implies that neutrophil degranulation is abolished by elevated blood glucose levels in inflammatory situations [142, 143]. Accumulating evidence in T2D subjects, the reduced phagocytic activity of PMBCs is significantly reversible if glycaemic management is improved. The reduced phagocytic activity in T2D patients can mostly be attributed to blood glucose management. A study has demonstrated that the anti-inflammatory drug propofol, when combined with a lipid emulsion prevented the formation of NETs by suppressing PMA-induced ROS [144]. High glucose induces the release of neutrophil elastase during NETs formation. In rodent models, silvestat, a neutrophil elastase inhibitor delivered via nanoparticles, prevented NETs formation, reduced clinical signs of lung damage, and lowers serum levels of NE and other proinflammatory cytokines [145]. Yang Liu et al. (2018) illustrated that intravenous injection of CRISPR-Cas9 plasmids encoding gRNAs that target NE were encapsulated into the cationic lipid-assisted nanoparticles (CLANpCas9/gNE) successfully diminished expression of NE in epididymal white adipose (eWAT) and in the liver, whereby they successfully mitigated the insulin resistance of T2D [146]. Prostaglandin E2 is a critical regulator of inflammation, inhibited NETosis by activation of the cAMP–PKA pathway through the activation of its Gαs‐coupled receptors, EP2 and EP4 [147]. Consequently, restoring neutrophil functions may serve as a therapeutic strategy to manage infections in T2D.

Nicotinamide mononucleotide (NMN) is an intermediate of NAD + biosynthesis, result of a reaction between a phosphate group and a nucleoside containing ribose and nicotinamide (NAM) [148]. Studies have shown that NAMPT-mediated NAD^+^ biosynthesis is severely conceded in metabolic organs such as liver and WAT of high-fat diet-fed mice (HFD). Strikingly, the administration of NMN a crucial NAD + intermediate and product of the NAMPT reaction improves glucose intolerance by restoring NAD^+^ levels in HFD-induced T2D mice. Further showed positive augments hepatic insulin sensitivity and activates SIRT1 which helps in restoring gene expression related to oxidative stress, inflammatory response, and circadian rhythm after NMN therapy [149]. A Randomized double-blind clinical trial of nicotinamide mononucleotide (NMN) therapy on postmenopausal overweight/obese women with prediabetes showed positive effects on Insulin-stimulated glucose disposal, insulin signaling, and muscle insulin sensitivity [150]. Study on HFD mice by Jun Yoshino et al., stated that administration of the NMN to diet and age-induced T2D mice can be an effective intervention to treat the pathophysiology of T2D. Recent studies showed that Sirtuin 1 (SIRT1) one of the mediators of NMN can be used as a target in T2D which will be a promising therapeutic target since it actively participates in regulating insulin resistance, inflammation, glucose/lipid metabolism oxidative stress, and mitochondrial function. which is one of the mediators for these beneficial effects of NMN [149, 151]. Deacetylation of SIRT1 regulates NF-κB which plays a major role in hepatic insulin resistance [152, 153] and a report by Yoshino et al., 2011 showed increased level of acetylated p65, a component of NF-κB in HFD-fed mice evidenced that SIRT1 activity was suppressed by HFD. Long-term NMN administration may be a highly effective strategy to maintain improved SIRT1 activity in tissues and organs [149]. Other sirtuin family members (SIRT2-7) also contribute to the metabolic effects of NMN. Deficits in NAMPT-mediated NAD + production may specifically impair the functioning of mitochondrial sirtuins (SIRT3-5), which may contribute to the mitochondrial dysfunction seen among T2D [154]. It would be interesting to find influence of NMN therapy on neutrophil (dys)function in T2D.

T2D is a major health concern worldwide. According to IDF Diabetes Atlas 10^th^ edition, it has been estimated that around 537 million people are suffering from diabetes globally, which will rise to 643 million (11.3%) by 2030 and to 783 million (12.2%) by 2045 with a huge mortality rate and more than 3.96 million people die worldwide every year due to T2D-associated complications including infections. Numerous theories have been put up to explain the relationship between diabetes and a higher risk of infections and many studies focusing on the possibly impaired neutrophil functions. However, mounting evidences confirm that glucotoxicity serve as a major cause for metabolic reprogramming of immune cells and render them incapable of effector functions. Collective data shows that metabolic routes like glycolysis, glutaminolysis and PPP are the major source of energy for the proper functioning of neutrophils which finds altered in diabetes individuals with infections. Therapeutic lowering of blood glucose may not be sufficient to manage T2D-associated infections due to the process of metabolic memory in different cell types. Shunting the metabolic pathways by treating with enzyme inhibitors may help in restoring NADPH pools to resensitize neutrophil functioning. Future studies are warranted to test these hypotheses in clinical models.

## Abbreviations

IL-6: Interleukin-6
6-AN: 6-Aminonicotinamide
2-DG: 2-Deoxyglucose
4OI: Itaconic acid
AGEs: Advanced Glycated End products
AKR1C1: Aldo–keto reductase family 1 member C1
AMPK: AMP-activated protein kinase
AMPs: Antimicrobial Peptides
ATP: Adenosine Triphosphate
BPI: Bactericidal/permeability-increasing protein
Casp6: Caspase 6
CCRs: Chemokine Receptors
CEBP-*α*: CCAAT Enhancer Binding Protein-Alpha
CEBP-*β*: CCAAT Enhancer Binding Protein-Beta
c-MPL: Thrombopoietin receptor
CRISPR: Clustered Regularly Interspaced Short Palindromic Repeats
CTLA-4: Cytotoxic T-Lymphocyte Antigen 4
CXCL3: Chemokine (C-X-C motif) ligand 3
DHAP: Dihydroxyacetone Phosphate
DPI: Diphenylene iodonium
EP2: Extracellular Protein 2
EP4: Extracellular Protein 4
ERK1/2: Extracellular signal-Regulated protein Kinases 1 and 2
eWAT: Epididymal White Adipose Tissue
FAO: Fatty Acid Oxidation
fMLP: F-Methionyl-Leucyl-Phenylalanine
G6PDH: Glucose-6-Phosphate Dehydrogenase
G-CSF: Granulocyte – Colony Stimulating Factor
GLUT: Glucose Transporter
GSH: Glutathione
HDP: Host Defence Peptides
HFD: High Fat Diet
HNP: Human Neutrophil Peptides
IFN-α: Interferon-Alpha
IL-1β: Interleukin-1Beta
LDH: Lactate Dehydrogenase
LILRB5: Leukocyte immunoglobulin-like receptor B5
LKB1: Liver kinase B1
LPS: Lipopolysaccharides
MMP: Matrix Metalloproteinases
MPO: Myeloperoxidase
NADPH: Nicotinamide Adenine Dinucleotide Phosphate Hydrogen
NE: Neutrophil Elastase
NECTIN2: Nectin cell adhesion molecule 2
NETs: Neutrophil Extracellular Traps
NF-κB: Nuclear factor κB
Nrf2: Nuclear factor erythroid 2-related factor 2
PAD4: Peptidyl Arginine Deiminase 4
PAI1: Plasminogen Activator Inhibitor 1
PARP: Poly (ADP-ribose) polymerase
PBMCs: Peripheral blood mononuclear cells
PFK: Phosphofructokinase
PKB: Protein kinase B
PLPP3: Phospholipid phosphatase 3
PMA: Phorbol 12 myristate 13 acetate
PPP: Pentose phosphate pathway
PRRs: Pattern recognition receptors
PTB-DM: Pulmonary tuberculosis with diabetes mellitus
RAGE: Receptor for advanced glycation end products
ROS: Reactive Oxygen Species
SCFAs: Short Chain Fatty Acids
SLC9A4: Solute carrier family 9 member A4
SOD: Superoxide Dismutase
T1D: Type 1 diabetes
T2D: Type 2 diabetes
TB: Tuberculosis
TCA: Tricarboxylic acid
TGF-α: Transforming Growth Factor-Alpha
TGF-β: Transforming Growth Factor-Beta
THP1: Tamm-Horsfall Protein 1
TLR-4: Toll-Like Receptors
TNF-α: Tumor Necrosis Factor-Alpha
